# Efficacy of linezolid on gram-positive bacterial infection in elderly patients and the risk factors associated with thrombocytopenia

**DOI:** 10.12669/pjms.293.2925

**Published:** 2013

**Authors:** Li-qing Bi, Jing Zhou, Ming Huang, Su-ming Zhou

**Affiliations:** 1 Li-qing Bi, Department of Geriatric Intensive Care Unit, the First Affiliated Hospital of Nanjing Medical University, Nanjing, China.; 2 Jing Zhou, Department of Geriatric Intensive Care Unit, the First Affiliated Hospital of Nanjing Medical University, Nanjing, China.; 3 Ming Huang, Department of Geriatric Intensive Care Unit, the First Affiliated Hospital of Nanjing Medical University, Nanjing, China.; 4 Su-ming Zhou, Department of Geriatric Intensive Care Unit, the First Affiliated Hospital of Nanjing Medical University, Nanjing, China.

**Keywords:** Elderly, Gram-positive bacterial infections, Linezolid, Thrombocytopenia

## Abstract

***Objective*** **: **Linezolid is active against drug-resistant gram-positive bacteria. However, the efficacy and safety of linezolid in the treatment of the elderly have not been well characterized. The purpose of this study was to evaluate the efficacy of linezolid in the treatment of the elderly with gram-positive bacterial infection and to investigate the risk factors associated with the development of thrombocytopenia in these patients.

***Methodology: ***This was a retrospective analysis of 50 elderly patients who were treated with intravenous linezolid for gram-positive bacterial infection. Clinical data and bacteriological responses were assessed. Risk factors associated with thrombocytopenia in elderly patients were analyzed.

***Results: ***The overall clinical cure rate of linezolid was 74%, and the bacteriological eradication rate was 69%. Thrombocytopenia occurred in 24 patients, and thrombocytopenia was associated with both the duration of treatment (*P *= 0.005) and the baseline platelet count (*P *= 0.042). Based on a logistic regression analysis, the baseline platelet count <200×10^9^/L (OR = 0.244; 95% CI = 0.068- 0.874;* P *= 0.030) was identified as the only significant risk factor for linezolid-associated thrombocytopenia in elderly patients. The mean platelet count decreased significantly from the 7^th^ day of treatment, and decreased to the lowest value 1-2 days after the end of therapy.

***Conclusions*** **: **Linezolid is effective and safe for the elderly with gram-positive bacterial infections. Adverse effects such as thrombocytopenia are of greater concern. Platelet counts should be monitored in patients who are treated with linezolid and that measures should be taken in advance to avoid hemorrhagic tendencies.

## INTRODUCTION

Gram-positive bacteria are important pathogens in community and nosocomial infections. In recent years, antibiotic-resistant gram-positive bacteria have caused a variety of diseases and deaths, among which methicillin-resistant staphylococcus aureus (MRSA) is a major pathogen.^1^ Linezolid was the first oxazolidinone to be developed and approved for clinical use in the USA in April 2000, and it is active against drug resistant gram-positive bacteria.^2^ Cross-resistance between linezolid and other protein synthesis inhibitors is really uncommon, and linezolid seldom induces drug resistance of bacteria in vitro.^3^^,^^4^ Therefore, linezolid is predominantly used in clinical treatment of bacterial infection. However, linezolid has been associated with hematologic adverse effects such as thrombocytopenia.^5^ At present, there are few studies that reported the efficacy and safety of linezolid in the elderly.

The purpose of this study was to evaluate the efficacy of linezolid in the treatment of the elderly with gram-positive bacterial infection and to investigate the risk factors associated with the development of thrombocytopenia in these patients.

## METHODOLOGY

***Patients and study design: ***This was a retrospective study that enrolled 50 patients with a mean age of 81 years (range, 60–96 years) who were treated with linezolid at 600 mg IV BID (q12h) between Jan 2008 and Oct 2010 at Jiangsu Province Hospital in Nanjing, China. Data were extracted from the electronic medical records obtained from the central database at the hospital. These patients were treated with linezolid as part of their primary antibiotic management of a suspected or proven Gram-positive infection, and have not used other drugs that clearly affect the platelet function.

The medical records of the study population were analyzed retrospectively. For each patient the following data were collected: demographics; length of hospital stay; intensive care unit (ICU) admission; type of infection and microbiological data; co-morbidities; previous and concomitant antimicrobial treatments; duration of linezolid therapy; outcome of linezolid treatment. The following laboratory findings before, during and after treatment were collected: hematologic properties (white blood cell count, hemoglobin, platelet count); routine biochemical tests; C-reactive protein (CRP); hepatic and renal function. The results of bacterial culture, smear, susceptibility tests and correlative imaging examinations were collected as well.

***Evaluation of the outcomes: ***According to the response to treatment, patients were classified as cured, failed or indeterminate. Clinical cure was defined as the resolution of the baseline signs and symptoms of infection with improvement or lack of progression of radiographic findings. Failure was defined as the persistence or progression of the signs and symptoms of infection after at least 7 days of therapy, administration of a potentially effective other antibiotics during the treatment because of lack of efficacy, or the absence of clinical assessments at the end of therapy. Indeterminate was defined as the inability to assign classification to one of these two categories.

Microbiological outcomes were classified as eradication, presumed eradication, persistence, eradication with reinfection. Microbiological response rate was defined as the number of patients with eradication or presumed eradication divided by the total number of patients in the analysis.

***Analysis of hematologic properties: ***Hematologic properties (white blood cell count, hemoglobin, platelet count) before, during and after the treatment were extracted from electronic medical records. All the data were statistically analyzed.

***Risk factors for thrombocytopenia: ***Thrombocytopenia was defined a decrease in platelet count of ≥25% and a final count of <100 × 10^9^/L. To investigate the risk factors associated with the development of thrombocytopenia in elderly patients who received linezolid therapy, the patients were divided into 2 groups according to whether thrombocytopenia occurred, and the following clinical characteristics were collected and compared in the thrombocytopenia group and the non-thrombocytopenia group: age, sex, length of hospital stay, baseline alanine aminotransferase (ALT) and creatinine clearance rate(CCr), baseline hemoglobin(HGB) concentration and platelet count, duration of linezolid therapy and lactic acid dehydrogenase(LDH) before the treatment.

***Statistical analysis: ***Results were expressed as mean values (±SD) unless otherwise specified. The unpaired Student’s *t *test was used to analyze continuous data, and either X^2^ analysis or Fisher’s exact test was used to analyze categorical data. Risk factors associated with thrombocytopenia were identified via logistic regression analysis. Statistical analyses were performed with SPSS for Windows, version 13.0 (SPSS). A *p *value of <0.05 was considered to be statistically significant.

## RESULTS

***Patient demographic and clinical characteristics: ***In total, 50 patients (36 men and 14 women, age≥60 years, mean age 81±10 years) were included in the study. Linezolid at the dose of 600 mg was administered by IV infusion q12h. Mean duration of treatment was 13±2 days (range 6-21 days), average hospital stay was 49±26 days. The clinical characteristics were shown in Table-I.

Mean planet count, hemoglobin concentration, liver and renal function and creatinine clearance rate of all patients were analyzed (Table-II). Based on t-test, a significant difference was observed only in the platelet count (*P*=0.000), whereas hemoglobin concentration, liver and renal function showed no significant differences before and after the treatment. In the 17 patients with renal dysfunction, renal function did not get worse after linezolid was used, and renal function of some patients improved with the improvement of their primary diseases.

A microbiologically documented diagnosis was made in 26 patients (52%). The most commonly isolated pathogen was *Staphylococcus aureus *(34%, of which 88% were MRSA), followed by *Staphylococcus epidermidis* (28%, of which 64% were MRSE), *Enterococcus faecium* (10%), *Enterococcus avium* (8%), *Staphylococcus lugdunensis* (4%), *Enterococcus faecalis* (4%), *Staphylococcus haemolyticus *(4%), *Staphylococcus capitis* (4%), *Staphylococcus hominis *(2%), *Staphylococcus sciuri *(2%). All isolates were susceptible to linezolid.

***Clinical and microbiological outcomes: ***Out of the 50 treated patients, 37 patients (74%) were considered to be cured. 5 patients were dead because of severe physical illnesses accompanied by respiratory and circulation failure. In the cured patients, the mean time to a decrease in temperature was 6±2 days, and the mean time to a decrease in white blood cell counts was 5±2 days. The bacteriological eradication rate was 69%(18/26).

**Fig.1:**
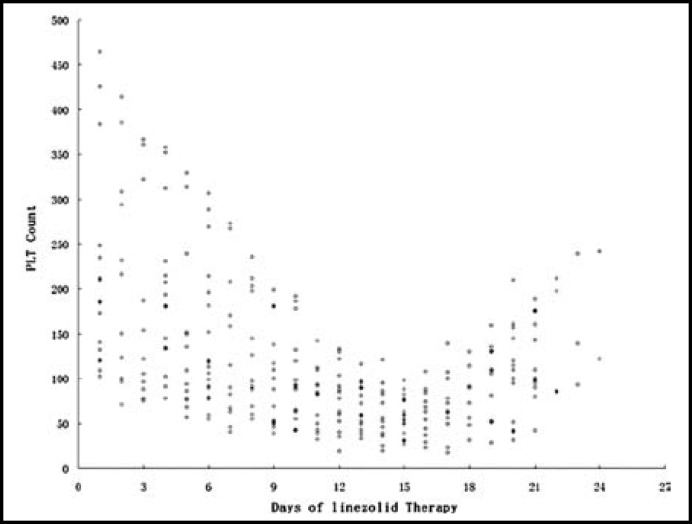
Platelet count (open circles,×10^9^/L) in patients of the thrombocytopenia group

**Fig.2:**
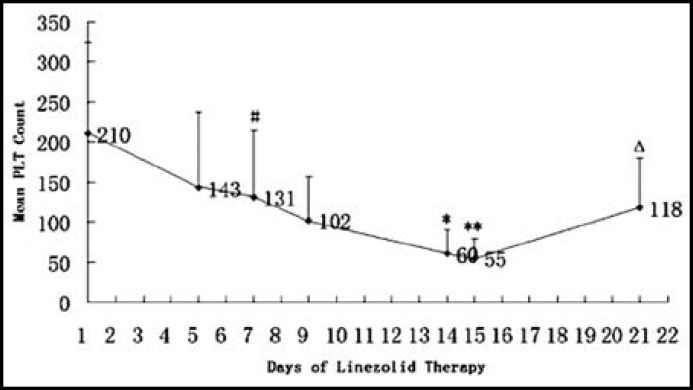
Mean platelet count in patients of the thrombocytopenia group

**Fig.3:**
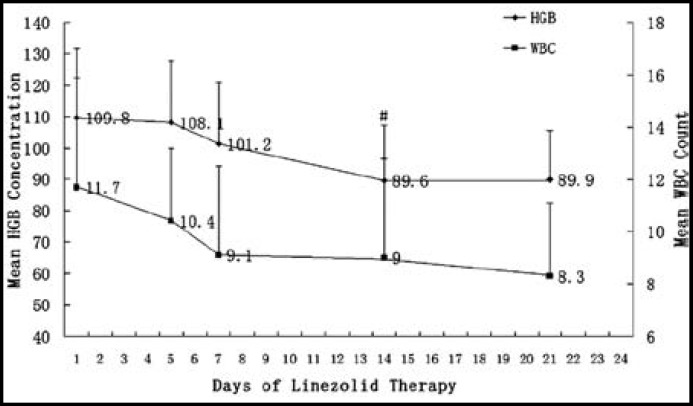
Mean white blood cell count (×10^9^/L, n=18) and hemoglobin concentration (g/L, n=18) in patients of the thrombocytopenia group

***Analysis of hematologic properties: ***Thrombocytopenia occurred in 24 patients, indicating the incidence of 48% (24/50). The platelet counts in all the patients in the thrombocytopenia group decreased since the beginning of linezolid therapy and decreased maximally at about day 15, then began to increase gradually (Fig.1). Among them, four patients accepted platelet transfusion because of the lower platelet counts and hemorrhagic tendencies, which happened on day 13, day 14, day 15 and day 17 of treatment, respectively. The platelet counts of most patients increased to near normal values 7 days after the end of therapy. The patterns of the changes in the platelet counts were demonstrated in Fig.2 of the patients who were treated with linezolid for 14 days, excluding those who accepted platelet transfusion. Simultaneously, the variations in white blood cell count and hemoglobin concentration in these patients were recorded (Fig.3).

Further statistical analysis showed that the mean platelet count of the patients was as follows: 210±114×10^9^/L (before treatment), 131±83×10^9^/L (d7), 102±55×10^9^/L (d9), 60±30×10^9^/L (d14), 55±24×10^9^/L (d15, 1 day after the end of therapy), and 118±62×10^9^/L (d21, 7 days after the end of therapy). We found a significant difference in platelet count between the time before treatment and 7 days after the therapy (d7,*P*=0.048). The difference became more significant at the end of therapy (d14, *P*=0.000), and the lowest mean platelet count were observed one day after the end of therapy (d15, *P*=0.000). On the other hand, the mean white blood cell count and hemoglobin concentration both decreased with the duration of linezolid therapy, and the hemoglobin concentration was significantly higher at the end of treatment than before the treatment (*P*=0.009).

**Fig.4:**
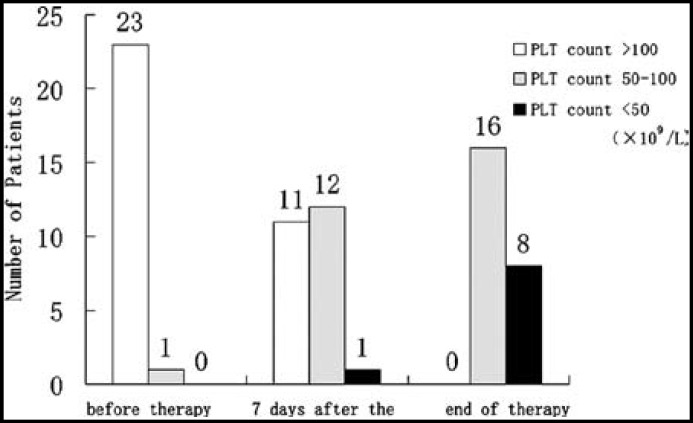
The population distribution classified by platelet counts in patients of thrombocytopenia group during treatment

In addition, we observed that in half of the 24 patients who developed thrombocytopenia, the platelet count decreased to below normal level when linezolid was used for one week (Fig.4).

***Risk analysis of thrombocytopenia: ***To investigate the risk factors associated with the development of thrombocytopenia in elderly patients who received linezolid therapy, the characteristics of patients were compared between those with and those without thrombocytopenia. As shown in Table-III, the mean (SD) treatment duration of linezolid was significantly longer in patients with thrombocytopenia than in those without thrombocytopenia (*P *= 0.005). In addition, the mean baseline platelet count was significantly lower in patients with thrombocytopenia than in those without thrombocytopenia (*P *= 0.042). Other risk factors such as age, sex, length of hospital stay, baseline alanine aminotransferase, creatinine clearancerate, hemoglobin concentration, lactic acid dehydrogenase were not significantly different between the 2 groups. However, based on a logistic regression analysis, the baseline platelet count <200×10^9^/L (OR = 0.244; 95% CI=0.068- 0.874;* P *=0.030) was a significant risk for linezolid-associated thrombocytopenia in elderly patients (Table-IV).

## DISCUSSION

Gram-positive bacteria, particularly multidrug-resistant *Staphylococcus aureus*, increasingly become the common causes of nosocomial and community-acquired infection.^6^ In USA, the rate of MRSA infection increased to 50%-60% according to data from the National Nosocomial Infections Surveillance System of the Centers for Disease Control and Prevention.^7^ Similarly, bacterial resistance monitoring in 9 major hospitals in China in 2006 showed that MRSA accounted for 58, 4% of infection by *Staphylococcus aureus*.^8^ In addition, the appearance of vancomycin resistant enterococcus (VRE), vancomycin intermediate S aureus (VISA) and vancomycin resistant S aureus (VRSA) brings difficulties to curing infectious diseases. Consistent with previous data, in this study we found that Staphylococci including MRSA and MRSE was the major pathogens isolated in the patients.

**Table-I:** Clinical characteristics of the study population

*Clinical condition*	*n*	*Percentage (%)*
*Types of infection*		
Pneumonia	33	66
Pneumonia concomitant with bloodstream infection	11	22
Pneumonia concomitant with urinary infection	1	2
Bloodstream infection	4	8
Empyema	1	2
*Characteristics associated with infection*		
Mechanical ventilation	30	60
Hematological disease	2	4
Renal inadequacy	17	34
Age >70 years old	43	86
Length of stay >30 days	38	76
Poor malnourished condition	24	48
Vessel in deep vein and urinary canal remaining	37	74
ICU admission	43	86

**Table-II:** Comparison of laboratory findings in the study population before and after treatment

*Indices*	*Mean value before treatment*	*Mean value after treatment*	*P*
PLT(×10^9^/L)	239±114	146±87	0.000
HGB(g/L)	102±18	94±16	0.052
ALT(U/L)	32±34	24±13	0.303
AST(U/L)	40±37	40±25	0.984	
Cr(μmol/L)	122±84	130±135	0.780
CCr(ml/min)	54±40	50±31	0.668

PLT, platelet; HGB, hemoglobin; ALT, alanine aminotransferase;

AST, aspartate aminotransferase; Cr, creatine; CCr, creatinine clearance.

**Table-III:** Comparison of patient characteristics between those with thrombocytopenia and those without thrombocytopenia who received intravenous linezolid therapy

	*Patients With* *Thrombocytopenia* *(n=24)*	*Patients Without* *Thrombocytopenia* *(n=26)*	*P*
Age (years)	83±9	80±11	0.344
Sex (no)			0.861
Male	17	19	
Female	7	7	
Length of stay (days)	47±29	51±32	0.658
Baseline ALT (U/L)	44±42	23±14	0.159
Baseline CCr (ml/min)	46±36	60±43	0.290
Baseline HGB (g/L)	104±21	99±16	0.358
Baseline PLT (×10^9^/L)	204±118	272±101	0.042
Treatment duration (days)	14±2	12±2	0.005
Baseline LDH (U/L)	289±182	272±168	0.815

**Table-IV:** Risk factors associated with the development of thrombocytopenia in elderly patients who received intravenous linezolid therapy (n = 50

	*OR*	*95% CI*	*P*
Age(>80 years)	1.091	0.300- 3.960	0.895
ALT(>50U/L)	0.420	0.079- 2.218	0.307
CCr(<30μmol/L)	1.064	0.220- 5.148	0.938
HGB(<90g/L)	1.444	0.309-6.734	0.640
PLT(<200×10^9^/L)	0.244	0.068- 0.874	0.030
Treatment duration (>14 days)	0.299	0.022-4.128	0.368

OR=odds ratio

Planet count was expressed as (x 10^9^/L, n=18).

#*P*<0.05, * *P*<0.01,**P<0.01 compared with mean PLT count before treatment.

Δ *P*<0.01 compared with mean PLT count (d15).

#P<0.01 compared with mean hemoglobin concentration before treatment.

Infection by drug resistant gram-positive bacteria such as MRSA could increase mortality rates and hospital charges and prolong the length of hospital stay. In 2003, Cosgrove et al. comprehensively analyzed previous studies and concluded that the fatality rate of MRSA infection was 20 times that of MSSA infection.^9^ Glycopeptide antibiotics are thought to be the drugs of choice in the treatment of MRSA infection, but failures in the treatment of pulmonary infections have occurred recently. The main causes of these failures are the poor lung tissue penetration of glycopeptide antibiotics and the drift of minimal inhibitory concentrations. In addition, glycopeptide antibiotics have a narrow therapeutic range and require therapeutic drug monitoring, particularly in patients with renal dysfunction.^10^ Potential adverse effects of vancomycin include renal toxicity and ‘red-man’ syndrome.

As the first oxazolidinone to be developed and approved for clinical use, linezolid blocks the first step of protein synthesis-initiation, unlike most other protein synthesis inhibitors which inhibit elongation. Due to this unique mechanism of action, cross-resistance between linezolid and other protein synthesis inhibitors is rare.^11^ With high penetration in tissues and fast efficacy for pulmonary infections, linezolid has become the recommended choice of treatment for vancomycin-resistant enterococcus infections, hospital- and community-acquired pneumonia, tuberculosis and meningitis.^12^^,^^13^ A two double-blind studies of patients with MRSA nosocomial pneumonia showed that initial therapy with linezolid was associated with significantly better survival and clinical cure rates than therapy with vancomycin.^14^ Similar clinical trial in China confirmed that the efficacy rate of linezolid was 78.6% in pneumonia patients.^15^ At present, the use of linezolid in the elderly remains limited and is mainly based on incidental case reports, with not enough information regarding its efficacy and tolerability in old patients. In the present study, the clinical data concerning the use of linezolid in 50 elderly patients were retrospectively analyzed. We found that linezolid was effective in the treatment of the elderly with gram-positive infection and the total clinical efficacy was 74%, similar to previous reports. In addition, the effective cases included not only bacterial culture positive patients but also smear positive patients, implying that linezolid can be used empirically in the following conditions: lack of bacterial culture; infection not controlled after initial antibiotic therapy; pathogenic bacteria in nubibus. Linezolid could be especially useful as appropriate empiric therapy for elderly patients with pulmonary gram-positive bacterial infection.

In our study, the main adverse reaction thrombocytopenia was reversible, in agreement with previous study.^16^ However, the incidence of thrombocytopenia was 48%, higher than what reported previously.^17^^-^^19^ The possible reason was that the selected patients in our study were the elderly, and the hematogenesis function of the elderly might be lower than in younger patients.

To investigate the risk factors associated with the development of thrombocytopenia, the patients were divided into 2 groups based on the occurrence of thrombocytopenia. In the thrombocytopenia group, we found that the platelet count decreased significantly on the 7^th^ day of treatment, and decreased to the lowest value 1-2 days after the end of therapy, then increased significantly one week later. In addition, thrombocytopenia was often accompanied by anemia. The pattern of changes in platelet count we observed is useful for monitoring of platelet counts when patients are treated with linezolid. The mechanisms underlying linezolid induced hematologic toxicity remain to be clarified, although bone marrow suppression may be involved. 

In our study, we observed the decrease of hemoglobin and white blood cell counts accompanying the thrombocytopenia, but we could not confirm the existence of bone marrow suppression due to the complicated influencing factors and the deficiency of the marrow smears. Based on a univariate analysis, we found that the mean treatment duration of linezolid was significantly longer and the mean baseline platelet count was significantly lower in patients with thrombocytopenia than in those without thrombocytopenia. However, based on a logistic regression analysis, only the baseline platelet count <200×10^9^/L was a significant risk for linezolid-associated thrombocytopenia in elderly patients. It has been reported that thrombocytopenia occurred when the time of treatment exceeded 14 days, implying that this side effect was associated with the accumulation of drug or metabolite.^20^

In the present study, the duration of linezolid treatment (>14 days) was not a significant risk for thrombocytopenia based on multivariate analysis. The difference in the patient population and relatively short duration may explain the inconsistent result. Because the activity of LDH in blood serum usually increased when platelet and red blood cells were destroyed, we collected the data of LDH levels before and after treatment and did not find evidence for the destruction of platelets. In addition, by statistical analyses, we found that thrombocytopenia was not associated with age, sex, length of stay, liver and renal function before treatment or baseline hemoglobin concentration. In a study of 331 patients in Japan, it was reported that the incidence of linezolid-associated thrombocytopenia was higher in patients with renal dysfunction (creatinine clearance < 50 ml/min) than in those with normal renal function.21 However, in our study, we did not find correlated factors except for the pretreatment PLT count and the duration of treatment because of the small patient population. Moreover, the relationship between the dose per body weight and the efficacy and safety of linezolid was not be precisely evaluated. Future studies involving larger samples may help identify and confirm the correlated risk factors.

During treatment with linezolid, we found that although platelet counts decreased below normal level, hemorrhagic tendency did not always occur, and platelet counts could increase gradually after the end of therapy. This proved that thrombocytopenia was a reversible adverse effect although platelet transfusion is necessary when hemorrhagic symptoms appear.

In conclusion, linezolid is effective and safe for the elderly with gram-positive bacterial infection. Linezolid causes minimal damage to the liver and renal function of the aged, but adverse effects such as thrombocytopenia should be monitored. Thrombocytopenia was associated with baseline PLT count and duration of treatment, and the baseline platelet count <200×10^9^/L was a significant risk for linezolid-associated thrombocytopenia in elderly patients. These findings suggest that platelet counts should be monitored in patients who are treated with linezolid and that measures should be taken in advance to avoid hemorrhagic tendencies.

## Authors Contributions

Liqing Bi: Research project: Conception; Statistical Analysis: Design, Execution, Review and Critique; Writing the first draft. Jing Zhou and Ming Huang: Research project: Execution; Statistical Analysis: Execution; Critical Review of Manuscript. Suming Zhou: Research project: Conception and Organization; Statistical Analysis: Review of Manuscript.
